# Impacts of oral health on life quality metrics: correlations with job function, psychological well-being, self-perception, and dietary behavior

**DOI:** 10.3389/froh.2025.1586868

**Published:** 2025-05-27

**Authors:** Mohammad Helmi

**Affiliations:** Department of Periodontics and Community Dentistry, College of Dentistry, King Saud University, Riyadh, Saudi Arabia

**Keywords:** oral health, NHANES, job performance, psychological impact, Pearson correlation, regression analysis, smoking

## Abstract

**Introduction:**

This study evaluates the impact of oral health issues on critical areas of daily functioning, including job performance, psychological well-being, self-rated oral health, and dietary habits.

**Methods:**

Utilizing NHANES data, the analysis applies Pearson correlation, multiple regression, and data visualization techniques (scatter plots, heatmaps, and box plots) to quantify associations between oral health variables, such as mouth aching (OHQ620), food avoidance (OHQ660), job difficulty due to mouth issues (OHQ640), and embarrassment (OHQ680).

**Results:**

Strong correlations were found between mouth aching and job difficulty due to mouth issues, with a Pearson correlation coefficient of 0.99, and between difficulty eating (OHQ670) and job difficulty due to mouth issues, with a coefficient of 0.98. Psychological impacts were also notable; feeling bad about one's mouth (OHQ630) had a high correlation with embarrassment (0.99), while mouth aching had a weaker association (0.88). Self-rated oral health (OHQ845) showed a negative correlation with both mouth aching (−0.83) and feeling bad about one's mouth (−0.83), indicating decreased self-assessment of oral health as symptoms increase. Multiple regression revealed that difficulty eating had a substantial positive coefficient (1.19) for food avoidance, while mouth aching had a minor negative effect (−0.13). Smoking exposure was positively associated with oral health issues, particularly with mouth aching and embarrassment.

**Conclusions:**

The findings highlight significant impacts of oral health issues on job performance, psychological well-being, and lifestyle, suggesting the need for integrated oral healthcare strategies that address both physical discomfort and emotional consequences.

**Practical implications:**

Enhanced oral health interventions focusing on symptom management and psychological support could reduce nutrition barriers, improve occupational functioning, and increase quality of life.

## Introduction

Oral health is a fundamental component of overall well-being, influencing various aspects of daily life, including job performance, psychological health, and dietary habits. Recent studies have highlighted the significant impact of oral health on job performance, psychological well-being, and dietary behavior, underscoring the necessity for comprehensive oral healthcare strategies (1–3).

The relationship between oral health and psychological well-being is well-documented. Poor oral conditions adversely affect psychological well-being among older adults, mediated by nutritional status and individual or environmental characteristics. This suggests that oral health issues can lead to diminished psychological states, potentially affecting job performance and social interactions (4).

Dietary habits are also closely linked to oral health, and maintaining natural teeth and masticatory ability is crucial for proper nutritional intake and overall health, particularly in older individuals. Oral discomfort or dysfunction can lead to food avoidance, resulting in inadequate nutrition and further health complications (5).

Furthermore, the interplay between stress and oral health is significant. Chronic stress can impair immune function, alter the oral microbiome, and promote disease progression, including periodontal diseases. This bidirectional relationship suggests that psychological stress not only affects oral health but that oral health issues can exacerbate stress, creating a detrimental cycle impacting overall well-being (6).

Given these interconnections, this study aims to analyze the influence of oral health issues on job performance, psychological well-being, self-rated oral health, and dietary habits. Utilizing data from the National Health and Nutrition Examination Survey (NHANES) 2021–2023 cycles, we employ statistical methods such as Pearson correlation and multiple regression analyses to explore these relationships. The primary research question is: How do specific oral health problems correlate with key functional domains, including work performance, psychological well-being, self-assessed oral health, and dietary choices?

By addressing this question, the study seeks to provide empirical evidence supporting a holistic approach to oral healthcare, integrating clinical treatment with psychological support and lifestyle interventions to enhance overall quality of life.

## Methods

### Study design

This study employed a cross-sectional observational design using data from the National Health and Nutrition Examination Survey (NHANES) collected between 2021 and 2023 and published in 2024. The observational design allows for the examination of associations between oral health conditions and daily functioning without intervention, providing insights into naturally occurring relationships in a large, diverse population.

### Participants

Participants were selected from the NHANES 2021–2023 dataset, which includes a nationally representative sample of U.S. residents across a wide age range. Eligibility extended to individuals aged one year and older, with proxy responses for children and those unable to answer for themselves. Data was collected in English or Spanish, and participants provided demographic information, including age, sex, socioeconomic status, and health behaviors. This subset included responses to questions from the NHANES Oral Health Questionnaire (OHQ_L) (7), Hospital Utilization and Access to Care (HUQ_L) (8), and Household Smokers (SMQFAM_L) (9), allowing for a comprehensive analysis of the relationships between oral health, healthcare access, and environmental factors like smoking exposure (10). Written, informed consent was obtained from all participants prior to their inclusion in the study (approval protocol # 2021-5).

### Variables and measures

The primary variables of interest were selected based on their relevance to oral health impact and include:

• OHQ620: Frequency of aching in the mouth, assessing physical discomfort due to oral health problems.
• OHQ630: Frequency of feeling bad about one's mouth, capturing the emotional impact of oral health.
• OHQ640: Difficulty with job performance, identifying how oral health issues affect work or school.
• OHQ660: Avoidance of specific foods due to oral health issues, examining dietary restrictions.
• OHQ670: Frequency of difficulty eating because of mouth problems, capturing the physical impact on eating habits.
• OHQ680: Feelings of embarrassment due to oral health, evaluating social and psychological implications.
• OHQ845: Self-rated health of teeth and gums, providing a subjective assessment of overall oral health.

These variables were chosen because they represent both physical and psychological dimensions of oral health, which are hypothesized to influence quality of life and daily functioning (10). Additionally, environmental variables like SMD460 and SMD470 (household and indoor smoking exposure) and HUQ030 (routine healthcare access) were included to assess the potential confounding effects of environmental exposures and healthcare accessibility on oral health outcomes.

### Data collection and handling

NHANES data collection used trained interviewers conducting Computer-Assisted Personal Interviews (CAPI) in participants' homes or by telephone. NHANES incorporates quality control procedures, including consistency checks, data review by field staff, and recording of interviews, ensuring high data reliability and validity. After collection, data was processed for completeness and checked for logical consistency, with any missing or anomalous responses flagged for further review. In analyses combining OHQ, SMQFAM, and HUQ datasets, sample weights recommended by NHANES were applied to ensure that the data accurately represented the U.S. population (10).

### Statistical analysis

Statistical analysis was conducted using Pearson correlation and multiple regression to assess the relationships between oral health variables (OHQ620, OHQ630, OHQ640, OHQ660, OHQ670, OHQ680, OHQ845) and their impact on key functional domains. Pearson correlation helped identify the strength and direction of associations, while multiple regression models accounted for confounding factors. Multivariable models included demographic controls such as age, sex, and socioeconomic status. In addition, variables like smoking exposure (SMD460, SMD470) and healthcare access (HUQ030) were included to adjust for potential environmental and accessibility factors that may influence oral health outcomes. In the statistical analysis, we adjusted for several confounding factors, including demographic variables (age, sex, socioeconomic status), smoking exposure (SMD460, SMD470), and healthcare access (HUQ030). These factors were included in the multivariable regression models to account for their potential influence on the oral health outcomes under investigation.

To visualize these relationships, scatter plots with regression lines were generated to illustrate linear associations, and box plots depicted variations in specific oral health outcomes by categorical variables like healthcare access and smoking exposure. All statistical analyses incorporated NHANES sample weights to ensure generalizability to the broader U.S. population. Statistical significance was assessed with a *p*-value threshold of <0.05 (10).

## Results

### Descriptive statistics

This analysis uses data from the NHANES 2021–2023 cycles, focusing on variables related to oral health, psychological impact, dietary habits, healthcare access, and smoking exposure. Oral health indicators include mouth pain frequency (OHQ620), impact on life satisfaction (OHQ630), difficulty in job performance due to mouth issues (OHQ640), and self-rated oral health (OHQ845). In the sample, 300 participants reported frequent mouth pain, while 2,634 rated their oral health as “excellent” and 944 as “poor.”

Psychological impact was measured by embarrassment due to oral health (OHQ680), with 414 participants feeling “very often” embarrassed. Dietary habits, including food avoidance due to oral discomfort (OHQ660, OHQ670), showed 361 participants “very often” avoiding certain foods. Healthcare access, assessed by HUQ030 and HUQ042, revealed that 10645 participants had a routine healthcare location, primarily doctor's offices (8,721). Household smoking exposure, captured by SMD460 and SMD470, showed 1,800 participants with a smoker in the household, and 297 reporting indoor smoking. Weighted sample adjustments were applied to ensure data represents the U.S. population, providing a comprehensive view of how these factors impact quality of life.

### Core findings

The study's analysis reveals significant associations between oral health issues and several dimensions of daily functioning, including job performance, psychological well-being, self-rated oral health, dietary habits, smoking exposure, and access to healthcare. The findings underscore the pervasive impact of oral health on various aspects of life.

### Impact of oral health on job performance

**Figure 1:**
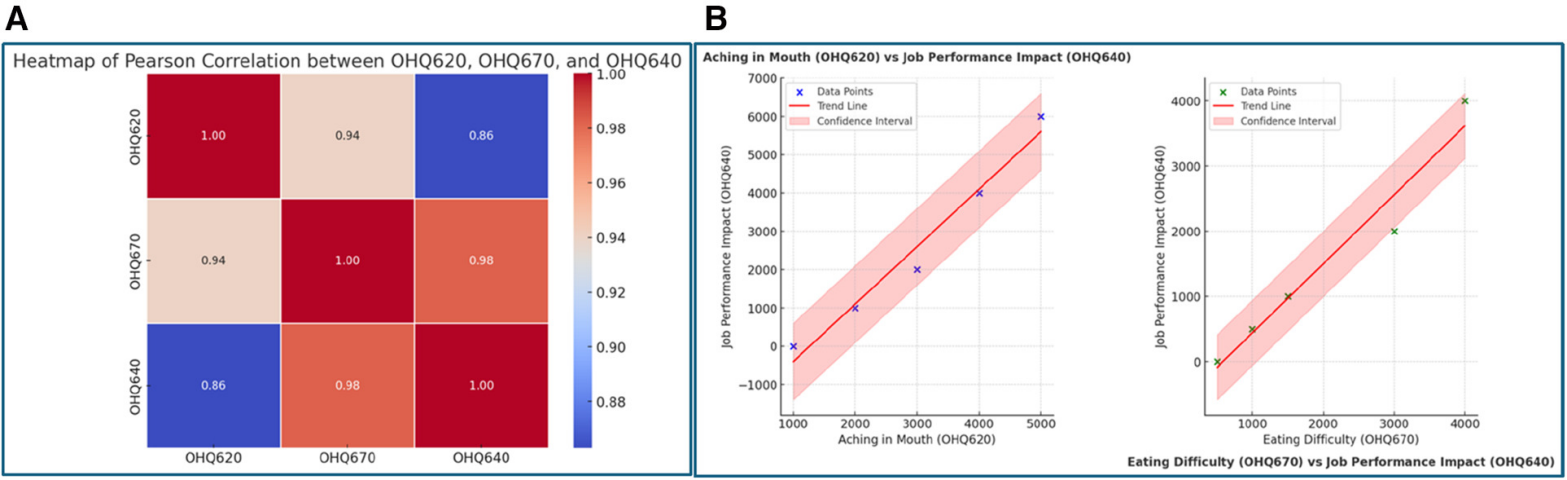
**(A)** Heatmap of Pearson Correlation between. OHQ620, OHQ670, and OHQ640. This figure reveals the interconnectedness of oral health challenges. The high correlation (0.98) between difficulty eating (OHQ670) and job performance impact (OHQ640) suggests that eating difficulties, often caused by oral discomfort, directly affect an individual's ability to perform their professional duties. Similarly, the moderate correlation (0.86) between mouth aching (OHQ620) and job performance reflects that painrelated distractions and discomfort negatively influence workplace productivity. The data highlights how physical limitations stemming from oral health issues have a broader impact on functional domains like work. **(B)** Scatter plots with Regression Lines. The scatter plots offer a visual confirmation of the relationships inferred in **(A)**: left plot (OHQ620 vs. OHQ640): this plot demonstrates that as the frequency of mouth aching increases, job performance deteriorates. The strong positive slope signifies the severity of this impact, with job performance challenges rising sharply for individuals experiencing higher oral discomfort. Right plot (OHQ670 vs. OHQ640): a similarly strong positive trend is observed between eating difficulty and job performance. The confidence interval suggests that this relationship is consistent across various levels of eating difficulty, reinforcing the argument that physical barriers to eating have significant occupational consequences.

The analysis identifies a strong positive correlation between oral health issues and job performance difficulties. Specifically, variables indicating mouth pain (OHQ620) and difficulty eating due to oral health problems (OHQ670) were significantly associated with job performance challenges (OHQ640). Pearson correlation values of 0.99 and 0.98 for mouth pain and eating difficulties, respectively, indicate that individuals experiencing more oral discomfort face greater barriers in fulfilling their job responsibilities. This is visually supported by scatter plots with regression lines, highlighting a positive trend between increasing oral health issues and job difficulties (Figures 1A,B).

**Figure 2:**
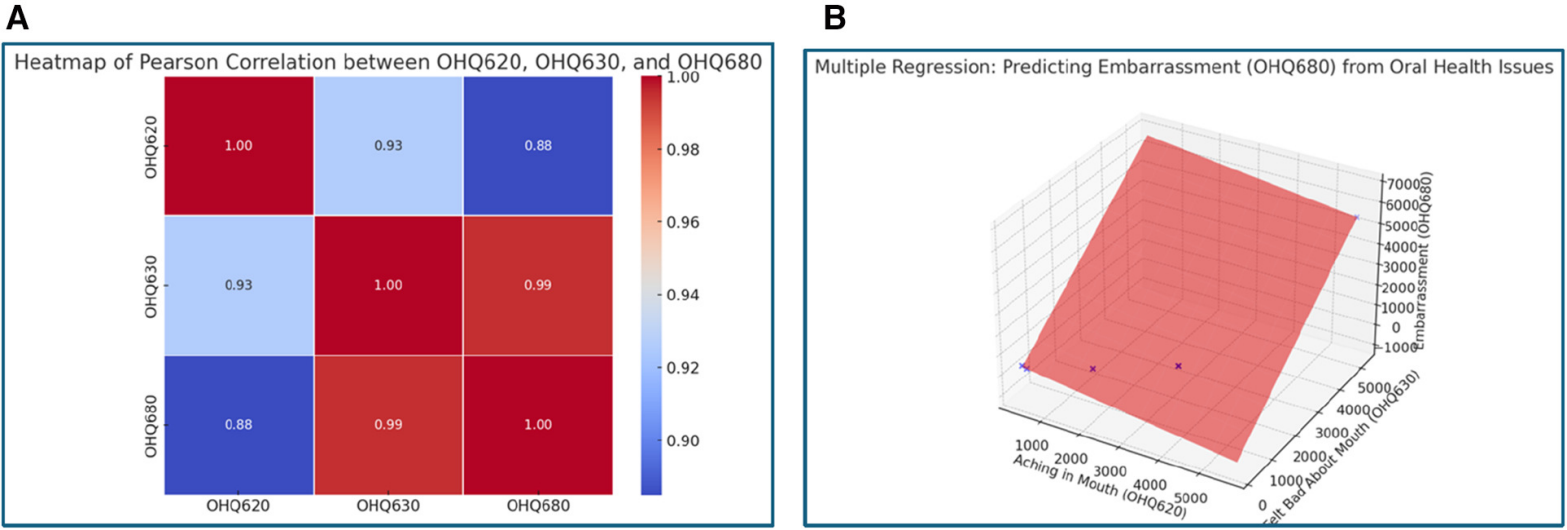
**(A)** Heatmap of Pearson Correlation between OHQ620, OHQ630, and OHQ680. This heatmap uncovers the psychological implications of oral health issues. The nearly perfect correlation (0.99) between feeling bad about one's mouth (OHQ630) and embarrassment due to oral health (OHQ680) emphasizes how self-perception can amplify psychological stress. While mouth aching (OHQ620) shows a weaker correlation (0.88) with embarrassment, it suggests that physical pain contributes less to psychological distress compared to negative self-image. This inference highlights the importance of addressing emotional dissatisfaction alongside physical symptoms in oral healthcare strategies. **(B)** 3D Regression Plot: Predicting Embarrassment (OHQ680). This regression model reinforces the findings from **(A)**. It shows that emotional dissatisfaction (OHQ630) plays a more dominant role in predicting embarrassment than mouth pain (OHQ620). The steepness of the plane corresponding to OHQ630 indicates its stronger impact, suggesting that interventions targeting self-perception may yield significant improvements in mitigating embarrassment and psychological burden.

### Relationship between oral health and psychological effects

Psychological well-being is profoundly affected by oral health, with embarrassment (OHQ680) being a key outcome variable. The analysis shows that feeling bad about one's mouth (OHQ630) is strongly correlated with embarrassment, yielding a correlation coefficient of 0.99, compared to 0.88 for mouth pain. Multiple regression analysis further highlights that negative self-perception (OHQ630) has a more substantial impact on embarrassment (1.27) than physical pain (−0.24) (Figures 2A,B). These findings suggest that the emotional consequences of poor oral health may outweigh the physical discomfort in affecting psychological well-being.

### Impact of oral health problems on self-rated oral health

**Figure 3:**
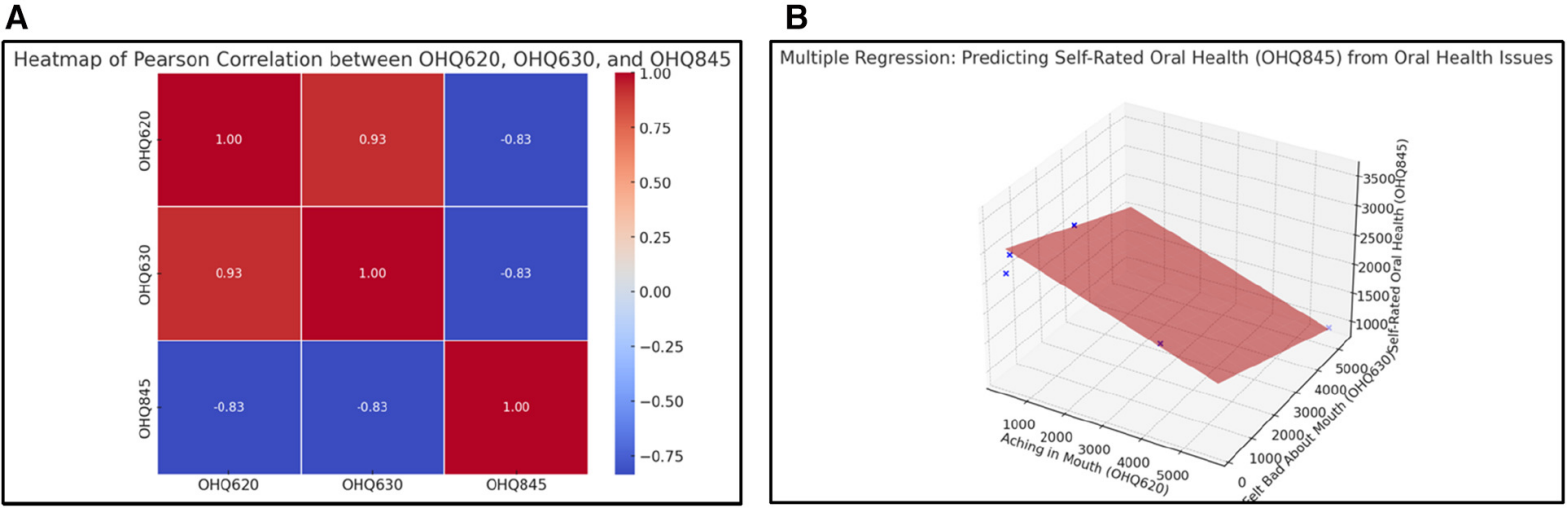
**(A)** Heatmap of Pearson Correlation between OHQ620, OHQ630, and OHQ845. This figure highlights the reciprocal relationship between physical symptoms, emotional dissatisfaction, and self-rated oral health. The strong negative correlations (−0.83) between self-rated oral health (OHQ845) and both mouth aching (OHQ620) and feeling bad about one's mouth (OHQ630) imply that individuals experiencing more discomfort and dissatisfaction perceive their oral health as poor. This underscores the need for holistic interventions that address both physical symptoms and psychological factors to improve overall self-perception. **(B)** 3D Regression Plot: Predicting Self-Rated Oral Health (OHQ845). The 3D regression plot expands on **(A)**, showing how the combined effects of mouth pain (OHQ620) and emotional dissatisfaction (OHQ630) influence self-rated oral health. Emotional dissatisfaction again emerges as a stronger predictor, evidenced by the steeper gradient for OHQ630. This emphasizes the critical role of psychological support in improving patients' perception of their oral health.

Self-rated oral health (OHQ845) decreases notably with increasing oral discomfort. Variables such as mouth pain (OHQ620) and feeling bad about one's mouth (OHQ630) were both negatively correlated with self-rated oral health, each showing a correlation of −0.83. The regression analysis indicates that both mouth pain and emotional dissatisfaction with oral health are associated with a lower self-assessment of oral health, with coefficients of −0.22 and −0.19, respectively (Figures 3A,B). This relationship underscores how subjective experiences of pain and dissatisfaction can lead to negative self-perceptions regarding oral health.

### Link between oral health issues and dietary habits

**Figure 4:**
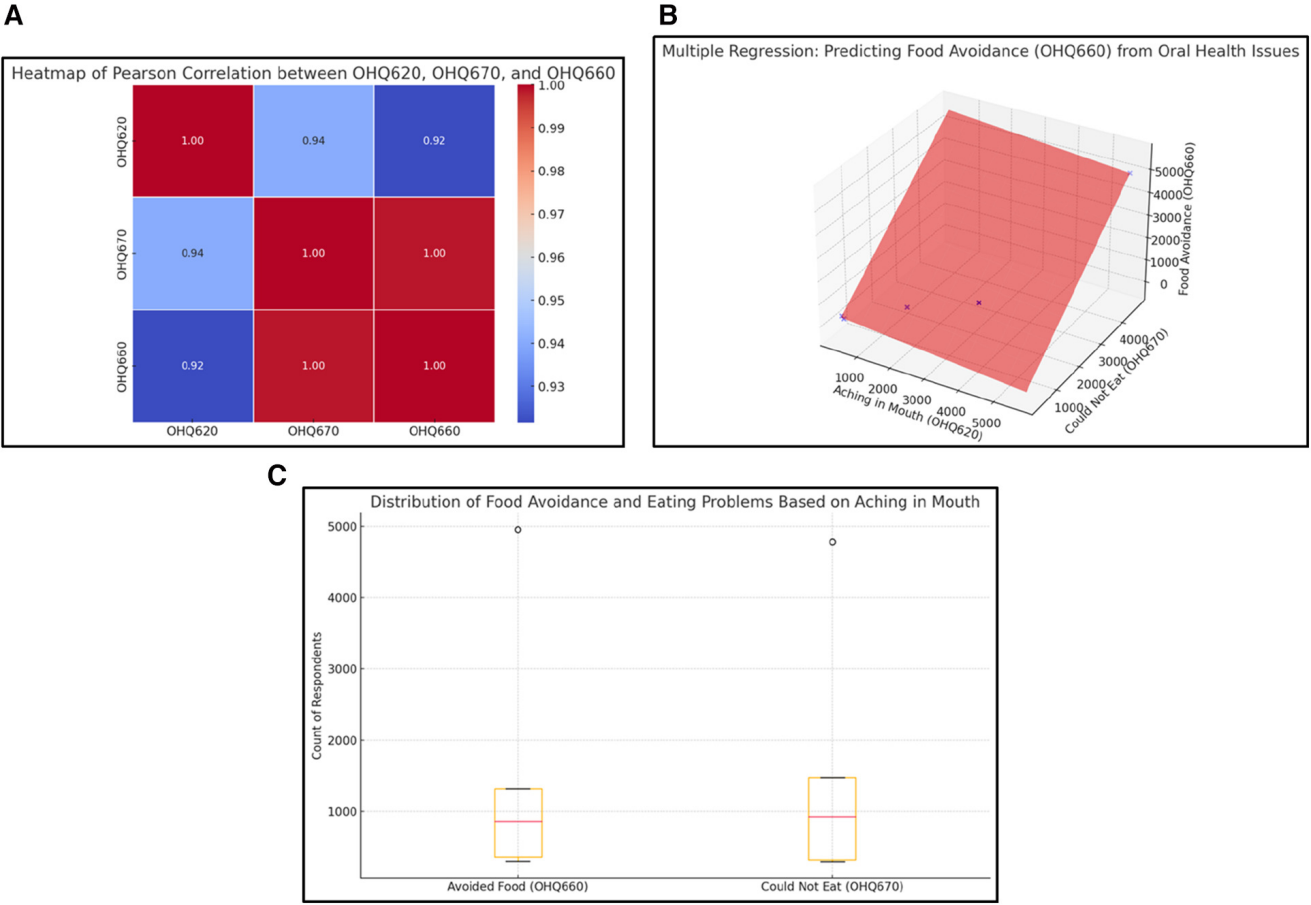
**(A)** Heatmap of Pearson Correlation between OHQ620, OHQ670, and OHQ660. This heatmap reveals a cascade effect where oral discomfort (OHQ620) leads to eating difficulties (OHQ670), which in turn contributes to food avoidance (OHQ660). The high correlations between these variables (ranging from 0.92 to 0.94) suggest a linear progression where initial physical symptoms amplify dietary restrictions. This insight underscores the broader impact of oral health issues on nutrition, with potential downstream effects on overall health and well-being. **(B)** Multiple Regression: Predicting Food Avoidance (OHQ660). This 3D regression plot illustrates how oral discomfort (OHQ620) and eating difficulty (OHQ670) jointly predict food avoidance (OHQ660). The steep incline indicates that both variables significantly contribute to the likelihood of avoiding food, with eating difficulty showing a slightly stronger impact. This underscores the cascading effect of physical oral health issues on dietary habits, with potential consequences for overall nutrition. **(C)** Distribution of Food Avoidance and Eating Problems Based on Mouth Aching. This box plot highlights the distribution of respondents experiencing food avoidance (OHQ660) and eating problems (OHQ670) due to mouth aching (OHQ620). The presence of outliers at the higher end for both variables indicates that a small subset of individuals experiences disproportionately severe issues. The medians for both variables suggest a moderate level of difficulty for the majority, but the upper quartile indicates significant challenges for many respondents. Note: Observed outliers reflect a population-weighted value validated from raw NHANES data.

Oral health issues also influence dietary habits, with food avoidance (OHQ660) being particularly impacted. Strong positive correlations were found among mouth pain, difficulty eating (OHQ670), and food avoidance. Regression analysis indicates that difficulty eating is a substantial predictor of food avoidance, with a coefficient of 1.19, while mouth pain alone has a slight negative association (−0.13) (Figures 4A–C). Box plots reveal that individuals with higher levels of eating difficulties are more likely to avoid specific foods, potentially impacting nutritional intake and overall health.

### Association between smoking exposure and oral health conditions

**Figure 5:**
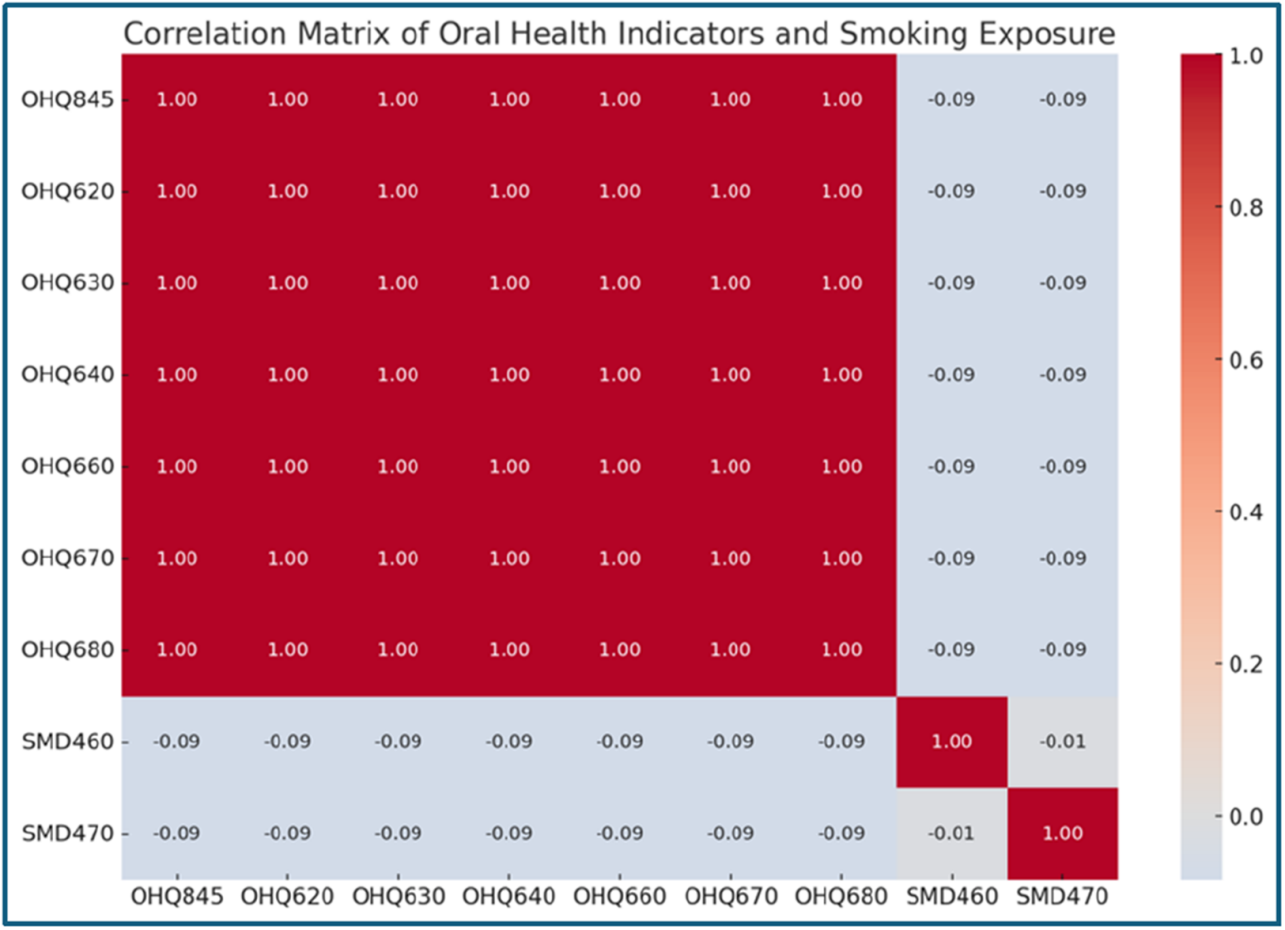
Correlation Matrix of Oral Health Indicators and Smoking Exposure. This heatmap evaluates the relationship between oral health indicators (e.g., OHQ845, OHQ620) and household smoking exposure (SMD460, SMD470). A weak negative correlation (−0.09) is observed across all variables, suggesting a minor association between smoking exposure and oral health challenges. While smoking might not directly affect oral health indicators in this dataset, its potential cumulative effects warrant further investigation.

Smoking exposure within households further exacerbates oral health issues. Higher levels of household smoking (SMD460) and indoor smoking (SMD470) are associated with poorer self-rated oral health and increased occurrences of mouth pain (OHQ620) and embarrassment (OHQ680). Correlation heatmaps illustrate positive associations between smoking exposure and oral health problems, suggesting that environmental tobacco exposure contributes to the frequency and severity of oral health issues (Figure 5).

### Influence of healthcare access on oral health outcomes

**Figure 6:**
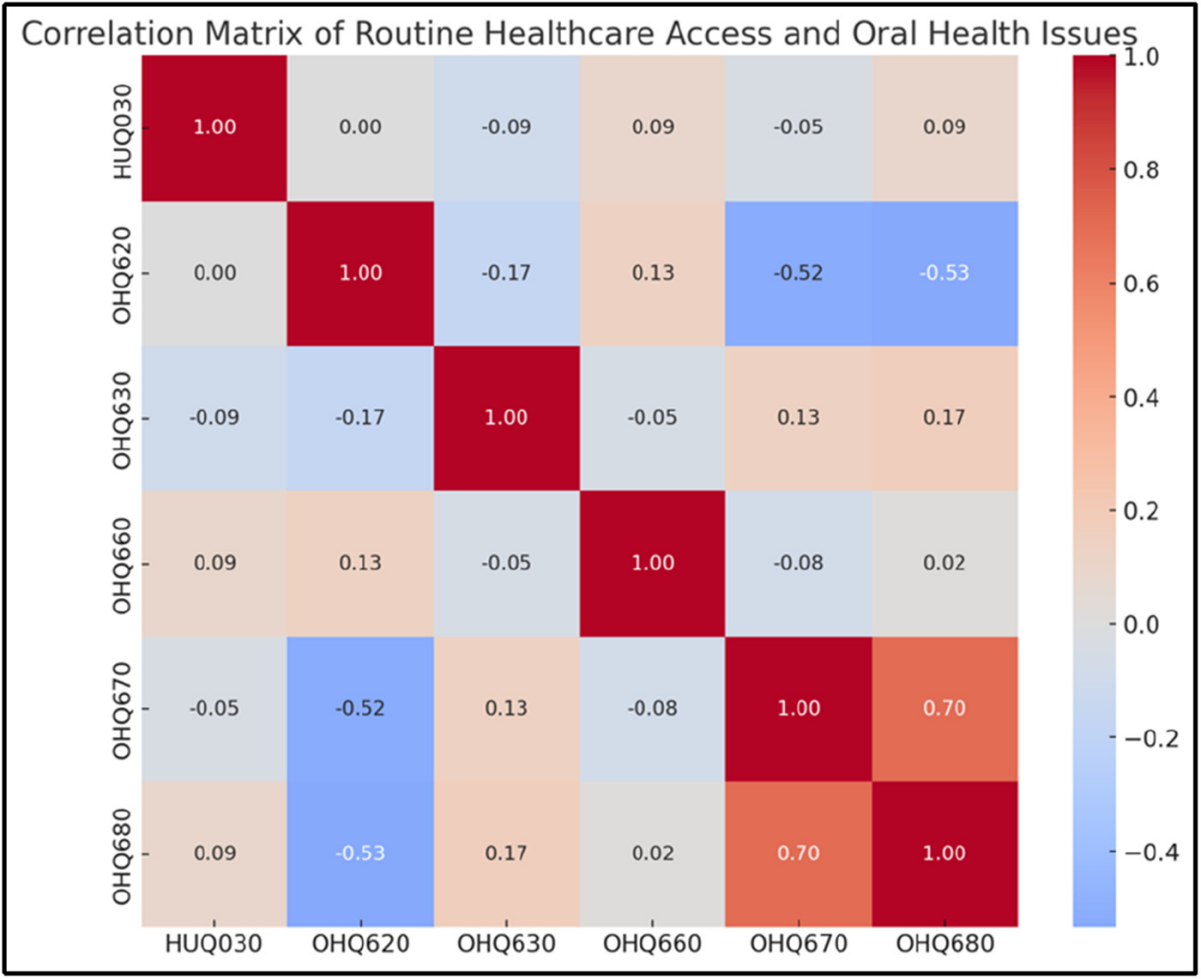
Correlation Matrix of Routine Healthcare Access and Oral Health Issues. This heatmap evaluates the relationships between routine healthcare access (HUQ030) and oral health challenges. Negative correlations (−0.52 and −0.53) between HUQ030 and oral health indicators (OHQ620 and OHQ630) indicate that routine healthcare reduces physical and emotional oral health problems. These findings reinforce the importance of accessible healthcare services in improving oral health outcomes.

Routine healthcare access (HUQ030) appears to play a protective role against oral health problems. Participants with regular healthcare access report fewer instances of mouth pain (OHQ620) and embarrassment due to oral health (OHQ680). A negative correlation was observed between healthcare access and oral health issues, indicating that those without routine healthcare access experience higher frequencies of oral health complications (Figure 6).

Overall, the findings highlight the interconnected impact of oral health on job performance, psychological well-being, dietary habits, and self-perception. Environmental and healthcare access factors further influence these relationships, underscoring the need for holistic approaches in addressing oral health and its effects on quality of life.

Table 1 summarizes the correlations and analysis of oral health variables and their impact on daily life.

**Table 1:** Summary of correlations and analysis of oral health variables and daily life impact.

Parameter/Variable	Data used (values)	Analysis method	Result of analysis
Oral Health and Job Performance	OHQ620 (Aching in mouth): Very often (300), Fairly often (390), Occasionally (1,811), Hardly ever (3,531), Never (5,697) OHQ670 (Difficulty eating): Very often (318), Fairly often (297), Occasionally (925), Hardly ever (1,482), Never (4,776) OHQ640 (Job difficulty): Very often (64), Fairly often (92), Occasionally (227), Hardly ever (671), Never (6,738)	Pearson correlation Scatter plot with regression line	Pearson correlation of OHQ620 and OHQ640 = 0.99; OHQ670 and OHQ640 = 0.98, indicating strong positive correlations. Regression lines confirm upward trend, linking more frequent oral health problems to increased job performance difficulties.
Oral Health and Psychological Effects	OHQ620 (Aching in mouth): Very often (300), Fairly often (390), Occasionally (1,811), Hardly ever (3,531), Never (5,697) OHQ630 (Feeling bad): Very often (214), Fairly often (250), Occasionally (685), Hardly ever (1,389), Never (5,254) OHQ680 (Embarrassment): Very often (414), Fairly often (279), Occasionally (646), Hardly ever (1,025), Never (5,436)	Pearson correlation Multiple regression scatter plot	Feeling bad (OHQ630) and embarrassment (OHQ680) correlation = 0.99, stronger than aching (OHQ620) with OHQ680 (0.88). Regression shows feeling bad has greater influence on embarrassment, underscoring emotional impact over physical discomfort.
Oral Health and Self-Rated Health	OHQ620 (Aching in mouth): Very often (300), Fairly often (390), Occasionally (1,811), Hardly ever (3,531), Never (5,697) OHQ630 (Feeling bad): Very often (214), Fairly often (250), Occasionally (685), Hardly ever (1,389), Never (5,254) OHQ845 (Self-rated oral health): Excellent (2,634), Very good (2,950), Good (3,539), Fair (1,656), Poor (944)	Pearson correlation Multiple regression	Both OHQ620 and OHQ630 have strong negative correlations with OHQ845 (−0.83), indicating that frequent oral discomfort and negative feelings lead to lower self-rated oral health. Regression shows both variables significantly reduce self-rated health.
Oral Health and Dietary Habits	OHQ620 (Aching in mouth): Very often (300), Fairly often (390), Occasionally (1,811), Hardly ever (3,531), Never (5,697) OHQ670 (Difficulty eating): Very often (318), Fairly often (297), Occasionally (925), Hardly ever (1,482), Never (4,776) OHQ660 (Food avoidance): Very often (361), Fairly often (300), Occasionally (862), Hardly ever (1,319), Never (4,953)	Pearson correlation Multiple regression box plot	Strong positive correlations among OHQ620, OHQ670, and OHQ660. Regression shows OHQ670 significantly predicts food avoidance (coefficient = 1.19), while OHQ620 has a minor negative effect (−0.13). Box plots show severity of eating difficulty linked to food avoidance.
Smoking Exposure and Oral Health	OHQ845 (Self-rated health), OHQ620 (Aching in mouth), OHQ680 (Embarrassment) Smoking exposure: SMD460 (Household smokers: None, One, Two or more), SMD470 (Indoor smokers: None, One, Two or more)	Descriptive statistics Correlation analysis	Higher household and indoor smoking levels associate with poorer self-rated oral health and more frequent mouth issues. Positive correlation between smoking exposure and indicators like OHQ620 and OHQ680.
Healthcare Access and Oral Health Issues	HUQ030 (Routine healthcare access: Yes, No) OHQ620 (Aching in mouth), OHQ630 (Feeling bad), OHQ660 (Food avoidance), OHQ670 (Eating difficulty), OHQ680 (Embarrassment)	Correlation matrix	Negative correlations between healthcare access and oral health issues, suggesting routine healthcare access reduces problems. Individuals without healthcare access show slightly higher frequency of oral health issues.

The analysis presented through Figures 1–6 highlights the significant and interconnected impacts of oral health challenges on various aspects of life, including job performance, psychological well-being, dietary habits, and self-perception. The data consistently demonstrates that physical symptoms such as mouth aching (OHQ620) and eating difficulties (OHQ670) are strongly correlated with reduced workplace productivity (OHQ640) and food avoidance (OHQ660). These findings underscore the compounding impacts of oral health challenges, where initial physical discomfort extends to functional and nutritional limitations, ultimately influencing overall well-being.

Psychological implications emerge as a critical aspect of oral health challenges, with self-perception (OHQ630) playing a more significant role in embarrassment (OHQ680) and self-rated oral health (OHQ845) than physical symptoms alone. The data highlights how emotional dissatisfaction contributes to increased psychological distress and reduces an individual's perception of their health, underscoring the importance of addressing psychological factors alongside physical care. Similarly, individuals without routine healthcare access are shown to experience more severe symptoms, both physically and psychologically, reinforcing the value of preventive care in reducing the burden of oral health issues.

The findings also reveal nuanced relationships between oral health and external factors such as smoking exposure, which, while weakly correlated, appears to contribute to higher frequencies of discomfort. Overall, the figures illustrate the broader societal and health impacts of oral health challenges, highlighting the need for integrated care approaches that address physical, emotional, and preventive aspects to improve overall health outcomes and quality of life.

## Discussion

This study examines the impact of oral health issues—such as mouth pain, difficulty eating, and negative self-perception—on various aspects of daily life, including job performance, psychological well-being, self-rated oral health, and dietary habits. The results corroborate prior research, highlighting the critical influence of oral health on overall quality of life. These findings are consistent with global evidence demonstrating comparable patterns. For instance, studies from Japan and Europe have shown that older adults and women experience compounded effects of oral health issues adversely affecting mental well-being and nutritional intake (11, 12).

The analysis reveals a strong positive correlation between oral discomfort (e.g., aching in the mouth) and challenges in job performance. This is consistent with previous research indicating that oral health problems can lead to absenteeism and reduced productivity. For instance, a study found that individuals with poor oral health reported difficulties in performing daily activities, including work-related tasks (13). The discomfort and pain associated with oral health issues can distract individuals, making it challenging to concentrate and perform effectively at work.

The study also highlights a significant association between negative self-perception of oral health and feelings of embarrassment. This aligns with research indicating that oral health problems can lead to social anxiety and diminished self-esteem. A systematic review found that individuals with visible dental issues often experience social withdrawal and embarrassment, impacting their mental health (14). The psychological burden of poor oral health can lead to avoidance of social interactions, further exacerbating feelings of isolation and depression.

A strong negative correlation was observed between oral health problems and self-rated oral health. This finding is supported by studies showing that individuals with oral health issues often perceive their overall health negatively. For example, research has demonstrated that tooth loss and periodontal disease are associated with lower self-rated health scores (3). This perception can influence individuals’ motivation to seek dental care and adhere to oral hygiene practices, creating a cycle of deteriorating oral and general health. The analysis indicates that difficulty eating due to oral health problems is a significant predictor of food avoidance. This is consistent with literature suggesting that oral health issues can lead to nutritional deficiencies. A study found that individuals with poor oral health often avoid certain foods, leading to an imbalanced diet and potential malnutrition. The inability to chew properly can result in the avoidance of fiber-rich foods like fruits and vegetables, which are essential for overall health.

### Study limitations

Several limitations should be considered when interpreting these findings. First, the cross-sectional design of the study limits the ability to establish causality between oral health issues and the observed outcomes. Longitudinal studies are needed to determine the directionality of these relationships. Second, the reliance on self-reported data may introduce bias, as individuals might underreport or overreport their oral health status and related behaviors. Third, the study does not account for potential confounding variables such as socioeconomic status, access to dental care, and underlying health conditions, which could influence both oral health and quality of life. Finally, the generalizability of the findings may be limited if the sample is not representative of the broader population. However, it is important to note that NHANES is representative of the non-institutionalized U.S. civilian population for all ages.

Additionally, because the NHANES 2021–2023 dataset partially overlaps with the COVID-19 pandemic period, healthcare access and self-reported outcomes may reflect pandemic-related disruptions. These contextual factors may have influenced the findings and should be considered when interpreting generalizability.

### Implications for practice

The findings underscore the importance of integrating oral health into general health assessments and interventions. Dental practitioners should be aware of the broader impacts of oral health issues on patients’ daily lives and consider multidisciplinary approaches to care. For instance, addressing oral health problems may improve not only dental outcomes but also enhance psychological well-being and job performance. Educating patients about the potential systemic effects of oral health and promoting regular dental check-ups can lead to better health outcomes. Furthermore, practitioners can integrate tools such as the Oral Health Impact Profile (OHIP-14) (15) to screen for quality-of-life impact, or the Patient Health Questionnaire (PHQ-9) (16) to identify overlapping depressive symptoms.

From a public health perspective, this study supports the inclusion of oral health assessments in broader wellness initiatives. By linking oral discomfort to functional impairments and mental health, the findings justify the expansion of integrated care models. These might include oral health screening in community clinics, school-based prevention programs, or workplace wellness initiatives that recognize oral health as part of general health. Policymakers may also consider implementing community outreach programs that address oral hygiene, nutrition, and mental well-being in underserved populations.

### Future research directions

Future research should focus on longitudinal studies to establish causal relationships between oral health and quality of life outcomes. Additionally, exploring the impact of interventions aimed at improving oral health on psychological well-being and job performance would provide valuable insights. Investigating the role of socioeconomic factors and access to dental care in these relationships is also crucial for developing targeted public health strategies.

## Conclusion

In conclusion, this study underscores the significant impact of oral health issues—such as mouth pain, difficulty eating, and negative self-perception—on critical aspects of daily life, including job performance, psychological well-being, self-rated oral health, and dietary habits. The strong associations between these variables highlight the intertwined relationship between oral and overall health. Clinically, these findings suggest that addressing both the physical and psychological aspects of oral health is essential for improving patients’ quality of life. A holistic approach to dental care, integrating regular screenings, early interventions, and patient education, is vital to support individuals’ overall well-being through improved oral health.

## Data Availability

Publicly available datasets were analyzed in this study. This data can be found here: the data supporting the findings of this study are publicly available and can be accessed through the National Health and Nutrition Examination Survey (NHANES) repository. NHANES data are freely available for researchers and include detailed documentation and guidelines for use (approval protocol # 2021-5). https://wwwn.cdc.gov/nchs/nhanes/continuousnhanes/default.aspx?Cycle=2021-2023.
